# Biomedical and Social Aspects of Spondyloepiphyseal Dysplasia Tarda Cases from Bengkulu District of Indonesia

**Published:** 2012-12

**Authors:** A. Ruyani, B. Karyadi, C. Muslim, Sipriyadi, Suherlan

**Affiliations:** 1 *Program Studi Pendidikan Biologi, Universitas Bengkulu, Jalan Raya Kandang Limun, Kota Bengkulu, Bengkulu 38371, Indonesia;*; 2 *Jurusan Biologi, Universitas Bengkulu, Jalan Raya Kandang Limun, Kota Bengkulu, Bengkulu 38371, Indonesia;*; 3 *Rumah Sakit Umum Daerah (RSUD) M. Yunus, the General Public Hospital of Bengkulu Province, Jalan Hibrida Sidomulya, Kota Bengkulu, Bengkulu 38225, Indonesia*

**Keywords:** male short stature, mutation, SEDL gene, spondyloepiphyseal, X-linked inheritance

## Abstract

**BACKGROUND:**

Although short stature male (SSM) cases are often found in South Bengkulu, no management reports about their existence are available. This paper summarizes the researches of biomedical and social aspects for the human genetic disorders.

**CASE PRESENTATION:**

Field survey results indicated that SSM community was located in Kedurang area, and 67 persons with SSM were successfully sampled from a population of 17,357 persons (one of 260). Anthropometric comparative studies, history of the pattern of X-linked inheritance, as well as the study of anatomy through radiology and ultrasound confirmed that SSM is spondyloepiphyseal dysplasia tarda (SEDT). Genomic studies through characterisation of mutations of the SEDL gene revealed that point mutations on SEDT Kedurang are different from the results of previous similar studies, and these people are predicted to come from the same ancestors. It is necessary to notice that persons with SEDT have normal intellectual ability, but the physical conditions make their socio-economic competitiveness very low. Furthermore premature joint pains make persons with SEDT become old faster than the ordinary people by the age of 40 years. Realizing that they are marginalized, some of them try to come together to establish a foundation designed to make a better life.

**CONCLUSION:**

It can be concluded that the appropriate management of SEDT should be done by integrating to improve their nutritional status, reduce the suffering of joint pain, develop labelled molecular markers for early detection, and increase their socio-economic competitiveness.

## BACKGROUND

Short stature (SS) is one of rare conditions of human nature. Several types of SS include spondyloepiphyseal dysplasia and acondroplasia. It is estimated that there are 200 kinds of SS more than 100 kinds are skeletal dysplasia (http://www.lpaonline.org). SS can be caused by many conditions and diseases, one of which is abnormal growth and development of bones (skeletal dysplasia). Spondyloepiphyseal dysplasia is characterized by growth abnormalities, spine abnormalities, and in some cases followed by vision abnormalities. The disorder is divided into two types, namely spondyloepiphyseal dysplasia congenital (SEDC) and spondyloepiphyseal dysplasia tarda (SEDT) (19). SEDT is a genetic disorder caused by abnormalities of the spine and the epiphyseal growth which is heterogeneous. It generally causes the growth failure of the spine, particularly the hip joint (18).

It is stated that individual with SEDT is seen with the following characteristics: 130-155 cm tall, short torso (not proportional), bent at the spine, bent at the shoulders, broad chest, short neck, wide face, flat jaw, normal-sized body member. The adult individual suffers from pain in the back, hips, shoulders, knees and joints (18). SEDT traits are not seen in ages of 5-10 years. These traits begin to appear when individuals reach 10-14 years old. SEDT is dwarfism generally suffered by men, because the pattern of inheritance is X-linked SEDT (interlock X chromosome) recessive (7). The properties of the X-linked are expressed more often in males than females, because females have a homologous pair of X chromosomes, unlike men who have only one X chromosome.

SEDT is caused by mutations in SEDL gene on the X chromosome, which is located on Xp22.2-p22.1 locus. The occurrence of gene mutations SEDL gene causes malfunction of cellular transport proteins in the cartilage. SEDT gene is a component of the particle grain carrier protein (TRAPP), a specific role in the transport from the endoplasmic reticulum to the Golgi vesicles (7). SEDL gene mutations causing SEDT cannot be identified with certainty, because it has very special characteristics. It has been known for four missense mutations causing SEDT as follows: three mutations (S73L, F83S, V130D) which map the inside of the protein. These mutations disrupt the structure of genes. The fourth mutation (D47Y) occurs on the surface, which can thwart the functional interaction with a partner protein (10). Furthermore it is suggested that the sedlin mutations S73L, F83S and V130D cause SEDT by sedlin misfolding, whereas the D47Y mutation may influence normal TRAPP dynamics (3). Identification of a TRAPPC2 mutation in a Korean pedigree revealed that a splice-donor site mutation in intron 3 of the TRAPPC2 gene (17).

**Figure 1:**
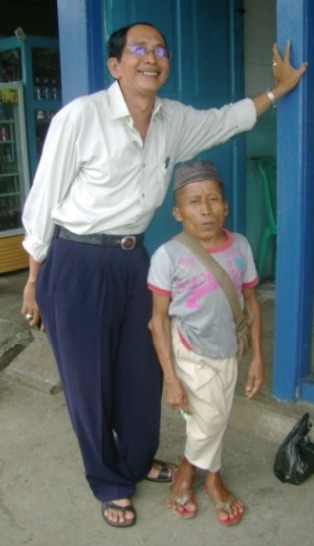
Physical profile of short statures male (SSM, right) are often found in South Bengkulu, Indonesia, couled with the normal male (left).

In Bengkulu short stature male (SSM) cases are very easy to find in South Bengkulu (Fig. 1). It still needs to be studied whether the SSM belongs to SEDC or SEDT. According to Durlani (2007; a retired paramedic) during the last thirty years, the number of SSM especially in Kedurang area has been increasing significantly. Since there is no adequate attention from the local health authorities, there have been no management reports about their existence. This paper summarizes the researches on biomedical and social aspects of the human genetic disorders in Kedurang.

## CASE PRESENTATION AND DISCUSSION

### Geographical location

Most SSM cases are often found in Kedurang subdistrict, in the northeastern South Bengkulu District, Bengkulu Province, Indonesia. Geographically its located at 4°29’49 87’’S 103°05’1190’’E. According to the National Land Agency of South Bengkulu the total area of Kedurang administration is approximately 23,455 hectares. Based on its altitude, Kedurang subdistrict is divided into four groups: 0-100 meters above sea level (asl; 50.88 percent of the area), 100-500 m asl (36.18%), 500-1000 m asl (5.94%), 1000+ m asl (7%). The population of Kedurang Subdistrict was 16.712 persons in 2002 and grew to 17.357 persons in 2006. The population distribution among villages is uneven. At the end of 2006, the subdistrict was divided into two subdistricts, namely Kedurang and Kedurang Ilir, which consists of 21 villages in 14 villages respectively. Most residents in these areas are traditional farmers who grow rice in rice field, and plant rubber, palm, or coffee in plantations.

Communities from the Kedurang river estuary up to the border between Kedurang and Kaur sub-districts speak Pasemah language which is derived from the Malay language. It is interesting that Kedurang communities usually use some words derived from English, among others: *sekul* for school, *poket* for pocket, blangkit for blanket, *rul* for ruler, *trai* for try, and stakin for socks. This fact indicates that Kedurang culture has long been in contact with the British colonial. There is information that the party of British Governor, Sir Thomas Stamford Bingley Raffles, had a long stay in Kedurang. Historical facts reveal that Bengkulu and British cultural communicated during the period 1685-1824.

### Field survey of SSM

Since the number of SSM cases in Kedurang needed to be determined exactly, we visited the location for several days to conduct a field survey. The field survey was aimed to know the distribution of SSM population in Kedurang subdistrict, South Bengkulu District, and the number of children from their marriage. The survey was conducted by interviewing respondents, using the structured questionaire which covered personal identification, age, education, job, and salary. The data were analyzed descriptively. This field of human genetics research required a good explanation, so some concerned people were voluntarily willing to help. The survey revealed that of the recorded 67 SSM people, 56 people would like to become respondents voluntarily (Table 1).

The 56 of SSM persons were distributed in 15 villages of Kedurang and 5 villages of Kedurang Ilir. These respondents could be divided respectively into the following age classes, namely (a) 15 years old, 11 people, (b) 16-18 years old, 2 people, (c) 19-30 years old, 16 people, (d) 31-39 years old, 8 people, (e) 40-50 years old, 13 people, and (f) 51 years old and older, 6 people. Range of their body height was 113.5-128.3 cm with an average of 119.2 cm (Table 1) (11). Married couples of SSM commonly have 3 children with a male and female ratio of 2:1. Their working performance is quite different from that of the normal person because of X form leg, and enlarged chest. Some mature SSM people suffer from envision abnormality, neural pain of the joint and spinal column. These physical limitations cause the average income of SSM families lower than that of normal people. Our interview indicated that income per month SSM persons was not more than 600,000 IDR.

**Table 1:** Distribution of SSM persons based on their age group, location, formal education, and height (cm) in the area of Kedurang who voluntary became respondents

No	Age group (year)	Phase of develop-ment	Number of SSM people in		Formal education					Total	Average Height (cm)
			Kd	KdI	Non	Ele.	Sec.	Hig.	Uni.		

1	-15	Puberty	9	2	1	5	5	0	0	11	113.5
2	16-18	Puberty	1	1	0	0	0	0	0	2	115.0
3	19-30	Adult	12	4	0	1	6	7	2	16	128.3
4	31-39	Adult	8	3	0	2	2	4	0	8	116.6
5	40-50	Late Adult	12	1	1	7	3	2	0	13	124.6
6	51-	Late Adult	6	0	1	2	1	2	0	6	117.3
Total/Average			45	11	3	17	19	15	2	56	119.2

Kd, Kedurang; KdI, Kedurang Ilir; Non, None; Ele., Elementary school; Sec., Secondary school; Hig., High school; Uni., University.

### Anthropometric comparation

Nineteen (19) persons with SSM which could be grouped into teenagers (those reaching puberty), adult, and late adult voluntary participated in this anthropometric comparative study. In this paper only a part of whole obtained anthropometric data are presented (Table 2) (14). Body height and vertebra length of SSM persons from all phases of development were lower than that of normal persons. Their body height was shorter than that of normal persons (136.5 cm), and the length of their vertebrae reached 51.5 cm, lower than the normal value (Table 2). In general orthogonal F tests showed that two indicators, body height and vertebra length, were significantly different between normal and SSM. This means, that statistically the body height and vertebra length of SSM at young, adult, and late adult are different from that of the same age group in normal condition (Table 2). Meanwhile it is indicated that SEDT tend to have body height of 129.5 cm (7), conforming what we observed in Kedurang which was less than 150 cm, and therefore it can be suspected that SSM is SEDT (14).

**Table 2:** Distribution of SSM persons based on body height (cm) and vetebra length (cm) in the area of Kedurang who voluntary became proband

Age group (year)	Phase of development	n	SSM		Normal	
			Average (cm)	Range (cm)	Average (cm)	Range (cm)

A. Body height						
12-15	Puberty	3	124.3 ± 6.8	118-130	147.5 ± 1,9	142-152
16-18		3	128.0 ± 10,8	117-134	160.5 ± 2.6	152-164
19-30	Adult	4	135.5 ± 5,3	129-142	165.5 ± 1.6	162-169
31-39		3	128.3 ± 8,6	120-135	163.4 ± 4.2	150-174
40-50	Late Adult	6	129.0 ± 6.0	120-141	161.2 ± 2.1	155-165
B. Vetebra length						
12-15	Puberty	3	45.0 ± 7.1	40-52	56.4 ± 1.0	54-59
16-18		3	49.7 ± 5.1	45-54	63.2 ± 1.3	61-68
19-30	Adult	4	51.5 ± 5.4	45-57	66.1 ± 1.9	63-71
31-39		3	48.0 ± 2.3	46-50	67.9 ± 2.3	61-73
40-50	Late Adult	6	50.2 ± 4.3	45-57	66.9 ± 1.1	65-69

n, number of probands.

The variation in body height is higher among persons with suspected SEDT than the normal ones. In terms of phenotypic expressivity, there are variations in the vertebrae, leg, and calf; but the rest is relatively invariable. Furthermore their penetrance are strong, mostly in the height, the length of vertebrae, and legs. Based on anthropometrical facts, it can be generalized again that the disorder in Kedurang belongs to SEDT (14). It is stated that final SEDT adult height is typically 137-163 cm which conforms the data from Kedurang that the body heights were between 117 to 134 cm among teenagers, 129-142 cm among adults, and 120-141 cm among late adults (Table 2). The data affirm that there are variations among the indicators of dwarfism at morphological phenotypes level, implicating the interaction between gene expression and environmental modifiers. Perhaps among the modifiers, nutrition is an important factor (14). It is widely accepted that genetic constituent is the main predictor for the body height, and is thought to make up more than 50% of the height.

### History of X-linked inheritance

**Figure 2:**
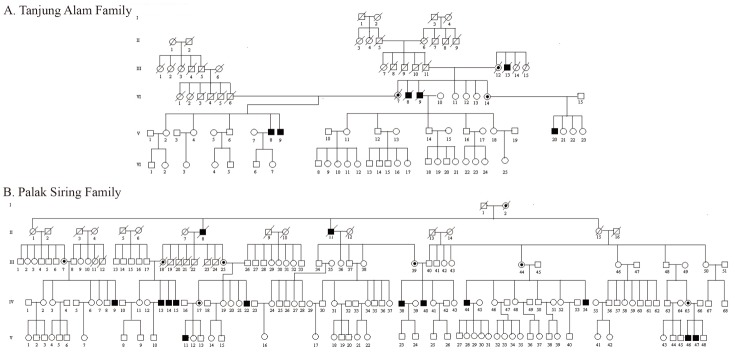
Although two of the eight SEDT families, Tanjung Alam (A) and Palak Siring (B) families, indicate the same pattern of inheritance at the closed area in Kedurang, but there is not yet enough kinship to combine the families. Individuals are represented as males (squares), females (circles), unaffected (open symbol), affected (filled in symbol) and obligate carrier (dot in middle of symbol). All other females are shown as unaffected (open circle).

There were eight (8) existing families having 32 suspected SEDT persons in Kedurang who voluntary participated in the study of inheritance history. In this paper only part of whole obtained inheritance historical data are presented (Fig. 2) (14, 15). In general pedigree trees reveal that when affected SEDT get married with a normal woman, they will get normal sons. One typical was found in first family; furthermore the same pattern always appears in all the family. It is clear that every dwarf man inherits his SEDT gene from his carrier mother (14). The suspected SEDT will not be get a SEDT son, but may be get SEDT grandson. As a result, it can be generalized that dwarfism belongs to a crisscross inheritance pattern and is believed to be SEDT caused by the mutation of SEDL gene located in the Xp22.2-p22.2 arm of X chromosomal as determined previously (4, 7).

The case of affected SEDT is always found in a normal family in which the mother is a carrier. The disorders in Kedurang are only found among the men with short body height which is significant after 10 years old, with shorter neck, bent shoulder, wider chest, obverse maxilla. During infant age their capability to walk develops slowly, and they suffer from rheumatic at elderly age. After examining the characteristics and analyzing the pedigree tree carefully, we can conclude again that SSM from Kedurang is affected SEDT having a thigh linked to X chromosome (14). The facts were similar to previous determination of affected SEDT. In order to understand the reliability of the phenotype ratio, we calculated the obtained data with Chi-Square test (20). Calculated X^2^ (0.72) indicated that the observed data are in line with the theory of genetic population. We also calculated the proportion of dwarf from generation to generation at maternal lines. Some probability of having children with affected SEDT varied among generation and family. The affected SEDT present in all generations with 55% to 70% possibility and in all families ranging from 46 to 83% chance (14).

Although eight SEDT families who have helped the exercise of this research seemed to indicate the same pattern of inheritance, but as a family they seemed to be separate from each other previously. Family relationships among them were known based solely on verbal information from parents who were still alive. It was not easy to track the kinship in Kedurang because they do not use the family name and there is not any established registration system for birth, marriage, and death. Relationship between Tanjung Alam and Palak Siring families can not be determined before the kinship positions are known (Fig. 2). Furthermore, genomic studies are really needed to ensure relationship among SEDT families in Kedurang or with the rest SEDT cases of the world.

### Anatomical studies

**Figure 3:**
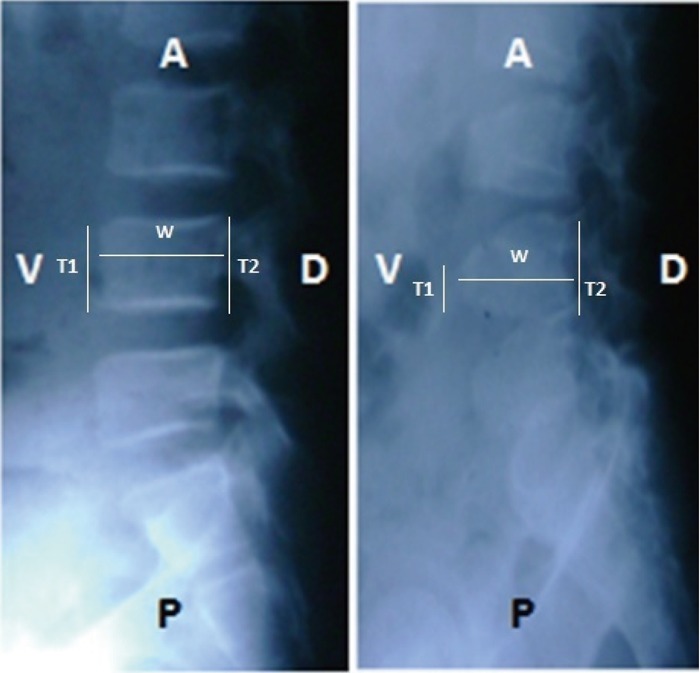
The size of ventral (V) part of lumber vertebrae on affected SEDT are significantly more thin compared to the dorsal (D) part which is possible related with early hunch-backed performance in some affected SEDT (right). This reality is very different compared to normal conditions (left). A=superior, P=inferior.

The closest hospital from Kedurang is a public hospital in Manna, South Bengkulu. Unfortunately both Rontgen and ultrasound equipment at the hospital were not adequate for anatomical study. A radiologist in our team suggested that affected SEDT should be invited to visit Bengkulu City with a hope the study could be conducted at the M Yunus Hospital. Furthermore the anatomical study could be conducted (2) successfully with the support of volunteers willing to travel 150 km from Kedurang to Bengkulu City. In this paper only a part of the anatomical data are presented (Fig. 3) (Table 3).

**Table 3:** Proportion of vertebra; ventral part (T1; cm) or dorsal part (T2; cm) to width (W; cm) at the lumbar vertebra-1, 2, 3, 4, and 5 (L1-5; cm) on the probands SEDT and normal (see Fig. 3)

Age group (years)	Status	n	Lumbal Vertebra (L _1-5_)									
			L _1_		L _2_		L _3_		L _4_		L _5_	

			T_1_/W	T_2_/W	T_1_/W	T_2_/W	T_1_/W	T_2_/W	T_1_/W	T_2_/W	T_1_/W	T_2_/W
Puberty (12-18)	Normal	1	0.68	0.71	0.67	0.67	0.67	0.67	0.69	0.71	0.69	0.71
	SEDT	2	0.61	0.40	0.65	0.47	0.67	0.36	0.65	0.43	0.68	0.49
Adults (19-30)	Normal	1	0.85	0.85	0.92	0.92	0.92	0.92	0.85	0.87	0.82	0.87
	SEDT	2	0.43	0.36	0.46	0,41	0.52	0.37	0.50	0.40	0.56	0.42
Late adults (>31)	Normal	1	0.86	0.86	0.94	0,86	0.97	0.97	0.86	0.86	0.83	0.97
	SEDT	5	0.47	0.35	0.49	0,36	0.50	0.29	0.54	0.36	0.51	0.37

n, number of probands.

Results of descriptive analysis of the anatomical data revealed that; 1) the vertebrae of affected SEDT decreased significantly (37.39-44.29%) compared to the unaffected; 2) the cubical chest of affected SEDT was anterior protruding of the ribs, barrel-shaped chest, caused by restricting of the thorax vertebrae; 3) the size of ventral part of lumber vertebrae on affected SEDT were significantly thinner than the dorsal part possibly related to early hunch-backed performance in some affected SEDT, platyspondyly (Fig. 3) (Table 3), and 4) the discus inter vertebra of affected SEDT decreased significantly (50-90%) compared to the normal. Ultrasound results illustrating the position of several organs in suspected SEDT are not presented in this paper. Based on the analysis of data descriptions of anatomy, it can be generalized that affected SEDT of Kedurang, South Bengkulu, morphological changes are caused by restricted inter vertebra segment and thinning discs. It can also be generalized that the bone morphology and the typography of liver and kidney on affected SEDT from Kedurang decrease as the effects of vertebrae and discus inter vertebral restriction (15).

### Genomic studies

Bengkulu University until now has not had sufficient laboratory equipment for both genomics and proteomics research. This fact encourages us to develop collaborations with established laboratories outside Bengkulu. Genomics research collaboration was conducted firstly with a zoology laboratory at Bagor Agricultural University in Bogor, West Java which produced some preliminary molecular data of SEDT (8). The second collaboration was implemented the following year with an established laboratory at the Oxford University, UK (16). The laboratory not only has a lot of experiences to do mutational analyses on the SEDT cases, but also stores enough information about the cases of SEDT in British society (4), and characterisation of sedlin mutations interactions with transcription factors MBP1, PITX1 and SF1 (10).

Sequencing of polymerase chain reaction (PCR) analysis on exon 4 of SEDL gene family at one short stature from Kedurang, South Bengkulu has been performed (8). Genomics study was conducted in Bogor with the stage of the work that has been standardized. The results of the alignment of each fragment of each sample showed no difference from the raw DNA sequence fragments. The fact indicated that there was no change of nucleotide meaning that there was no sequence base mutation in exon 4 of the SEDL gene.

The results of genomic studies in the UK concerning characterization of mutation of the SEDL (TRAPPC2) gene in patients with X-linked SEDT from Bengkulu District of Indonesia indicated that the same mutation was identified in each of the three probands studied and occurred at the splice acceptor site of intron 2 of the *SEDL* gene. This mutation is the first mutation described in Indonesian families (16). There were two important results from genomic studies in the UK, namely: (a) affected SEDT in Kedurang was thought to have come from the same ancestors, and (b) there were no sufficient facts to state that SEDT Kedurang has kinship with effected SEDT from UK or elsewhere in the world.

### Educational experiences

Affected SEDT are not facing just a medical problem but also psycho-social ones. Some of them have low self-esteem in adolescence, so they can not act optimally in school or community (1). There is not yet any real government and foundation policy with the mission to help affected SEDT. School drop-out rate is high enough for affected SEDT. How to motivate their enthusiasm for learning is not a simple effort. The facts indicate that the physical limitations lower their social and economic competitiveness in the agricultural community. Early joint pains suffered by all affected SEDT consequently cause them to be old faster, at the age of 40 years. They need specific skills training for increasing their economic productivity. The expression slogan of optimism “living comfortable with SEDT” requires a long holistic research.

The results of case studies regarding achievement motivation of students with SEDT at high and secondary schools in Kedurang, among others, indicated that students with SEDT ever feel ashamed, inferior, and were reluctant to come to school knowing their physical conditions were not the same as those of other students. Achievement motivation and learning achievement in biology for all affected SEDT were not the same. Family economic circumstances strongly influenced the high or low achievement motivation of students with SEDT. SEDT parents should be able to give special attention to children who have genetic disorders by way of encouragement, advice to their children not to feel inferior and ashamed of their physical conditions. SEDT children need to feel worthy to perform better. It is neccessary to recommend that SEDT students should go to vocational school SEDT, so they have skills that correspond to the weak physical state for life fulfilment in the future (1).

There is apparent evidence of affected SEDT in both school and community which demonstrate that they have normal intellectual ability, and this reality is similar with the previous report (18). In general their lack of education is due to limited purchasing power of their families. An affected SEDT who come from well-established economy could finish his undergraduate physics program, and then he is currently continuing his study on the Graduate School of Education Science at Bengkulu University.

### A foundation for SEDT community

**Figure 4:**
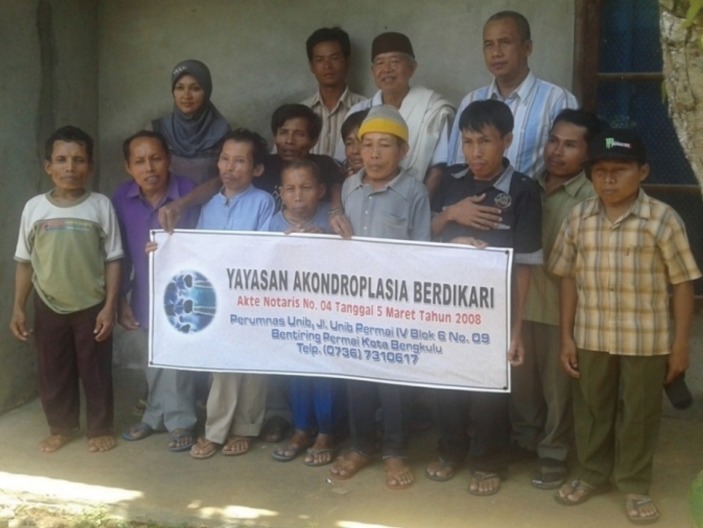
An awareness of the marginalized people, some of them tried to come together in YAB for designing a better life. Several advisors and board of the foundation after their coordinative meeting in Bengkulu.

Affected SEDT and their family are aware of the physical limitations that must be faced with a positive effort to plan for a better life. Closed attitude, low self-esteem, and not accepting the reality will only make them more marginalized in their community. They are also aware that the genetic disorder is inherited from the same ancestors. This awareness stimulate some of them to feel that coming together into a foundation is absolutely necessary for increasing the quality of life. With the full support of the team from Bengkulu University, on March 5, 2008 a foundation, namely Yayasan Akondroplasia Berdikari (YAB) (Fig. 4) was formally founded before a notary in Bengkulu City. An optimistic slogan “living comfortable with SEDT” is the brief aim of the foundation which will be achieved.

After the establishment of the foundation nearly four years ago, not much have been performed for the welfare of affected SEDT and their families in Kedurang. Only several activities have been carried out such as leaf-cutting equipment procurement, practical training of organic fertilizer production (12), business of pepper mill service, sending one person affected SEDT in the practical training in Java, and initiating development of joint venture company in poultry. During the period of four years the activities were depended on limited source of funding which could be collected by the team from Bengkulu University. More extensive cooperation with either governmental or non-governmental agencies is necessary to obtain additional sources of funding. Meanwhile it is also necessary to increase internal work ethic and to improve the foundation management.

### Future challenges

Almost all publications concerning SEDT stated that the human genetic disorder is rare in the world (5, 11) In Britain for example, the incidence of SEDT is one of the 500,000 (4). Meanwhile the results of our survey revealed that SEDT population was 67 persons from 17.357 which is equivalent with one in 260. That number is believed to be higher than with all the reports SEDT ever published. In the future there is possibility that SEDT population will continue to grow. Here are some of the challenges for affected SEDT from Kedurang to be addressed based on the priority scale. First, biomedical aspects, namely: (a) improve the nutritional status of affected SEDT to make better fit with the provision of nutrition, (b) medical treatment to reduce the suffering of joint pain that occurs in almost all affected SEDT, and (c) develop labelled molecular markers (nucleic acid) for early genomic detection (Southern blot analysis] (13, 18) as an effort of Eugenics in order to decrease the risk of SEDT.

Second, psycho-social aspects, namely: (a) plan a rational and simple explanation about the genetic disorders for affected SEDT and their family, (b) increase the positive view of given genetic reality and achievement motivation for a better life, and (c) provide scholarship assistance for affected SEDT and their family. Third, socio-economic aspects, namely: (a) optimize the role of the developing foundation as a forum for communication among affected SEDT and their family, (a) provide life skills training to increase their socio-economic competitiveness, and (d) incubate joint venture company to create self income generator for affected SEDT and their family.

## CONCLUSION

It can be concluded that the appropriate management of SEDT in Kedurang, South Bengkulu should be conducted by integrating improve their nutritional status, reduce the suffering of joint pain, develop labelled molecular markers for early detection, and increase their socio-economic competitiveness.

### Consent

Written informed consent from the patient for publication of this case report and any accompanying images. A copy of the written of consent is available for review by the Editor-in –Chief of this journal.

### Competing interests

The authors have declared that no competing interests exist. The funders had no role in study design, data collection and analysis, decision to publish, or preparation of the manuscript.

### Authors’ contributions

AR-contributed to both biomedical and social aspects, conducted collaboration with a laboratory at the Oxford University, and conceived the manuscript. BK-contributed to social aspect. CM-contributed to biomedical aspect. Sip-consulted the families, and performed to prepare molecular testing. Suh- contributed to biomedical aspect and performed Rontgen and ultrasound studies.
